# Asymmetric Reciprocal Crossing Behavior of an Andean Blueberry (*V. meridionale*) × Lingonberry (*V. vitis-idaea*) Hybrid

**DOI:** 10.3390/plants11223152

**Published:** 2022-11-18

**Authors:** Mark K. Ehlenfeldt, Elizabeth Ogden, Lisa J. Rowland

**Affiliations:** 1 Philip E. Marucci Center for Blueberry and Cranberry Research and Extension, USDA-ARS, Chatsworth, NJ 08019, USA; 2 Genetic Improvement of Fruits & Vegetables Laboratory, USDA-ARS, Beltsville, MD 20705, USA; elizabeth.ogden@usda.gov (E.O.); jeannine.rowland@usda.gov (L.J.R.)

**Keywords:** tetraploid, polyploid, septaploid, *Pyxothamnus*, *V. corymbosum*, *V. virgatum*, imprinting, EBN, endosperm balance number, genome strength

## Abstract

The fertility and crossing behavior of a tetraploid hybrid of 4x Andean blueberry (*V. meridionale*) and lingonberry (*V. vitis-idaea*) was evaluated through a series of crosses. Crosses of the hybrid with highbush blueberry produced divergent results. When used as a female with *V. corymbosum* males, virtually all offspring were hexaploid, most likely arising from 2*n* = 4*x* = 48 female gametes, and 1*n* = 2*x* = 24 male gametes. However, when used as a male, tetraploid hybrids were produced, resulting from 1*n* = 2*x* = 24 gametes from each parent. To further examine this crossing behavior, the 4*x V. meridionale*—*V. vitis-idaea* interspecific hybrid was pollinated with 6*x V. virgatum* (rabbiteye blueberry). Analogous to the previous crosses, 7*x* hybrids were produced from the joining of 2*n* = 4*x* = 48 female gametes with 1*n* = 3*x* = 36 male gametes. Such reciprocal crossing asymmetry is unprecedented. The ability to produce both 6x and 4x offspring from the same *V. corymbosum* parents allows the potential of bridging a *V. meridionale* hybrid genotype to both the tetraploid (*V. corymbosum*) and hexaploid (*V. virgatum*) commercial crop levels.

## 1. Introduction

*V. meridionale* is a species of section *Pyxothamnus* known mostly from northern Venezuela, westwards into northern Colombia, and then disjunct to the island of Jamaica. It is widespread in its range but only locally common. Its elevation range stretches from ca. 1000 to 2800 m, where it can form a shrub up to 3.5 m tall.

*V. meridionale* is characterized by small leaves and by racemose inflorescences with as many as 15–25 flowers per inflorescence. Notably, flowering material has been collected nearly every month of the year. Its fruit is usually spherical, approximately 14–20 mm in diameter, dark reddish to dark maroon–black to blue–black, and is very uniform in size when optimally pollinated.

Our original interest in *V. meridionale* was for its high number of flowers per bud as well as their loose inflorescence structure that might make hybrids with highbush blueberry amenable to machine harvest [1]. Plants of *V. meridionale* have the potential to develop an upright, tree-like bush structure with a monopodial base, furthering their potential value for breeding for mechanical harvest. The fruit is relatively thick-skinned, reflective of their high levels of anti-oxidants [2], and has subsequently been recognized to possess partial internal fruit pigmentation and an extremely small fruit attachment scar, <1 mm (Ehlenfeldt, personal observations).

In our earliest crosses, *V. meridionale* (at that time identified as *V. corymbodendron*) was crossed with diploid species from several taxonomic sections of Vaccinium and was found to produce almost exclusively triploid offspring [3]. It was recognized that *V. meridionale* had either a “genome strength” effect that rendered it compatible with some diploid species or was immune to the typical genome dosage effects that regulate the success of many interspecific crosses. In this first cycle of crossing, an anomalous tetraploid hybrid of 4*x V. meridionale* × 2*x V. vitis-idaea* (lingonberry) was produced by the functioning of a 2*n* gamete. That hybrid was self-fertile and through selfing gave rise to a small group of self-hybrid offspring [4]. Among these, several plants exhibited greater vigor, prolificacy of flowering, and fertility. US 1930, US 1933, and US 1993, in that order, were found to be the most vigorous and fertile genotypes among these offspring [4].

Ehlenfeldt and Luteyn [5] subsequently documented the ability of 4*x V. meridionale* to hybridize directly with 4*x V. corymbosum* (highbush blueberry) to produce fertile offspring, and Ehlenfeldt and co-workers [6] demonstrated the production of similar fertile 4*x* hybrids through direct hybridization of 4*x V. meridionale* with colchicine-derived 4*x V. macrocarpon* (cranberry).

Cultivar development in blueberry is a time-consuming process, with the development time of conventional crosses typically taking 10–15 years or more [7,8]. The development of useful breeding parents from species materials is can be equally time-consuming, typically requiring 1–2 backcrosses or intermatings to recover a near-commercial phenotype. When introgressing beyond the secondary gene pool, crossability and fertility issues also need to be investigated. In the course of exploring the means for the introgression of *V. meridionale*—*V. vitis-idaea* germplasm into useful forms for the breeding of *V. corymbosum*, we hybridized the best among of our *V. meridionale*—*V. vitis-idaea* hybrids with *V. corymbosum* and *V. virgatum* and evaluated offspring ploidy and fertility.

## 2. Results

The three hybrids that we used, US 1930, US 1933, and US 1993, are tetraploid self-progeny of US 1184 (=*V. meridionale* NC 3737 × *V. vitis-idaea* ‘European Red’) that have been discussed previously, and their behavior in crosses with lingonberry have been documented [4]. Although the three genotypes are siblings and all have the same genetic background, US 1930 distinguished itself as the most vigorous and fertile (as both male and female) of the three clones and thus became the focus of the majority of our crossing.

**Figure 1:**
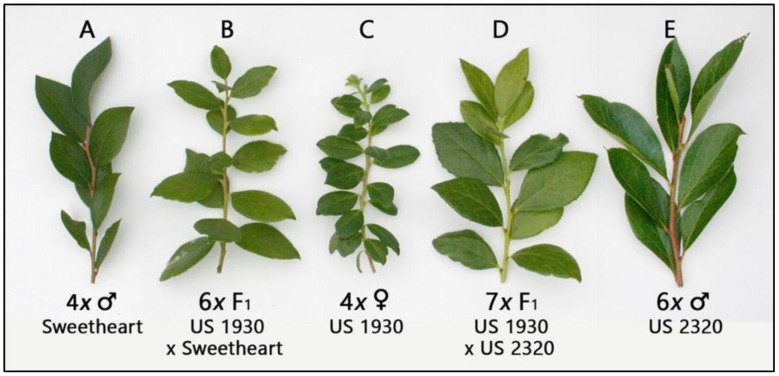
Leaves of 4*x* US 1930, cultivar parents, and their hybrids (L to R): (**A**) 4*x V. corymbosum* ‘Sweetheart, (**B**) 6*x* US 2540 (=4*x* US 1930 × 4*x* ‘Sweetheart’), (**C**) 4*x* US 1930, (**D**) 7*x* US 2521-A (=4*x* US 1930 × 6*x V. virgatum* US 2320), and (**E**) 6*x* US 2320 *V. virgatum* (=‘Nocturne’ × ‘Florida Rose’).

In our approach to unusual germplasm, our consistent goal is to incorporate the material into a form compatible with commercial 4*x V. corymbosum*. Thus, our first crosses with US 1930, US 1933, and US 1993 used these hybrids as females in 4*x* × 4*x* crosses, so any hybrids would unquestionably be offspring of the *V. meridionale—V. vitis-idaea* hybrids. Over a period of 2 years, these 4*x* genotypes were pollinated with a range of different 4*x* highbush cultivars and selections (Table 1). Fertility was only modest at best, and in over 290 pollinations, seeds were produced at a level of 0.71 seed/pollination (s/poll.)—1.6 seed/fruit (s/f). When planted out, these seed produced plants that were sufficiently unlike the female parents to be reasonably assumed to be hybrids. Many of these seeds produced plants that grew slowly and poorly. Many of these plants failed to reach heights of more than 5 cm before dying. One of the best of these hybrids plant-wise is pictured in Figure 1; however, this plant has not yet flowered. As some plants achieved a size at which it was felt foliage could be sacrificed, tissue was collected for flow cytometry. Of 17 plants that were tested, 14 were determined to be hexaploid, and 3, all from a cross of US 1930 × ‘Dixieblue’, were found to be intermediate, estimated to be either 5*x* (2 plants) or approximately 5.75*x* (1 plant). One of the hybrid plants from the cross US 1930 × ‘Sweetheart’ grew sufficiently to flower at a low level and was designated as US 2386.

Hexaploid US 2386 was crossed as a female to two 4*x V. corymbosum* cultivars (‘Camellia’ and ‘Chandler’) with the goal to initiate a return to a 4*x V. corymbosum* compatible level. In these pollinations, crosses succeeded at a level of 10.1 s/poll. (11.2 s/f) (Table 1). This fertility level is nearly 10-fold higher than the original crosses. A random sampling of 20 6*x* US 2386 × ‘Camellia’ seedlings from a population of >170 verified these plants as pentaploids (range 2.66–3.11 pg DNA vs. 2.96 pg mid-parent value from 4*x* and 6*x* standards).

Since US 2386 was a hexaploid, this second-generation plant was also crossed to a 6*x V. virgatum* hybrid (ARS 07-97) (Table 1). In these crosses, seeds were produced at a rate of 7.0 s/poll.—28.0 s/fruit. Much like the crosses to 4*x V. corymbosum*, this fertility level was greatly improved over the original cross success, and is nearly 17.5-fold higher than the original.

When it was determined that the original 4*x* F_1_ × 4*x* highbush crosses produced hexaploids, we presumed they arose most likely from the gametic fusion of a 2n female gamete with a 1n male pollen nucleus (2*n* = 4*x* = 48 + 1*n* = 2*x* = 24) since the *V. corymbosum* cultivars were expected to be meiotically regular. We hypothesized by analogy that crosses of 4*x* US 1930 × 6*x V. virgatum* would produce septaploids (i.e., 2*n* = 4*x* = 48 + 1*n* = 3*x* = 36). In the first cycle of such crosses, we made 121 pollinations, and produced 31 ‘good’ seed at a rate of 0.26 s/poll.—0.6 s/f (Table 2). From these, nine plants were produced that have grown to adequate maturity for ploidy determinations. All of these plants proved to be the hypothesized septaploids (Figure 1).

Septaploids might be expected to be fertile due to higher ploidy and polyploid buffering [9,10,11,12,13,14]. Tests of one of these septaploid genotypes found sufficient female fertility that 25 pollinations yielded 83 potentially viable seeds at 3.3 s/poll.—4.6 s/f. In a second cycle of such crosses, we made 212 pollinations, and produced 71 ‘good’ seeds at a similar success rate of 0.33 s/poll.—0.6 s/f. These plants have not yet been ploidy tested.

We next sought to determine whether this same result occurred in reciprocal crosses. In crosses of four different 4*x V. corymbosum* females by 4*x* US 1930, and one combination of *V. corymbosum* × US 1933, 54 pollinations produced 74 ‘good’ seeds at a rate of 1.37 s/poll. (or 1.6 s/f) (Table 4). Only four of the five cross-combinations produced plants. Among the progeny of ‘Sharpblue’ × US 1930 and ‘Cara’s Choice’ × US 1930, a sampling of 16 and 17 plants, respectively, were verified as 4*x* by flow cytometry. These plants were relatively slow growing in their juvenile period and moderately susceptible to powdery mildew in the greenhouse but were dramatically superior to the hexaploids produced in the 4*x* F_1_ × 4*x* highbush crosses.

One plant among the ‘Sharpblue’ × US 1930 progeny distinguished itself by growth rate and vigor and was subsequently numbered as US 2537-A. Tetraploid US 2537-A was crossed as a female with 4*x V. corymbosum* cultivars, and has produced relatively large sized seed of excellent quality. In these crosses with ‘Duke’, ‘Magnolia’, and ‘Talisman’ it has produced 11.8, 23.4, and 3.9 s/f respectively (Table 3).

Analogous to the crosses of 4*x V. corymbosum* × 4*x* F_1_s, we made the reciprocal crosses of 6*x V. virgatum* and 6*x V. virgatum* hybrid females by US 1930. In these crosses, seeds were produced at a rate of 0.58 s/poll.—1.3 s/f. From these crosses, two confirmed pentaploids were produced. In appearance, these plants did not look unambiguously hybrid; however, this may be due both to the relative F_1_ genome dosage 2*x* F_1_ sets: 3*x V. virgatum* sets, as well as the mixed genetic background of the 6*x* parents.

## 3. Discussion

The ploidy asymmetry seen these reciprocal crosses is unprecedented in any genus or commodity. While the current results may seem highly specific, the implications for germplasm utilization, speciation, and gene flow are profound.

Our goal in crosses such as these with US 1930 and its siblings was to incorporate and/or make this germplasm compatible with 4*x V. corymbosum*. What is notable is the counterintuitive results obtained. Routine crossing with a 4*x* hybrid would suggest that the cross should be executed as we originally attempted, a 4*x* F_1_ × 4*x V. corymbosum*, and indeed, this cross succeeded at a low level, although limited at least to some degree by the frequency of 2*n* gamete production on one parental side. The unexpected result was that the progeny of these crosses were all hexaploid or in a few cases pentaploid, but at any rate, not tetraploid. A hexaploid arising from a 4*x* × 4*x* cross suggested that one of the two parents produced a 2*n* gamete. When rationalizing our particular crosses, logic suggested that our 4*x V. corymbosum* male most likely produced normal functional 1*n* = 2*x* = 24 gametes, and logic also suggested that an exotic hybrid might have meiotic and/or fertility problems and might produce 2*n* gametes at some level. The initial conclusion was that our hexaploid progeny had two chromosome sets each of *V. meridionale*, lingonberry, and highbush blueberry. The negative aspect was that these hybrids proved to be rather weak and grew poorly. Nonetheless, one of these hybrids produced a small number of flowers, was evaluated for further fertility, and succeeded in crossing with both tetraploid and hexaploid partners. The value of these sets of advanced hybrids is undetermined, but what is notable is that this second-generation F_1_ hexaploid hybrid (US 2386) exhibited improved levels of fertility (as determined by seeds per fruit) in both of these combinations.

Because of this unexpected result, we needed to determine if the reciprocal of this cross behaved similarly. Surprisingly, it did not. The reciprocal cross behaved in the expected manner of a 4*x* × 4*x* cross, producing 4*x* hybrids. These hybrids did not use the same *V. corymbosum* parental genotypes as the hexaploid-generating crosses (Table 1); however, both sets of progeny generated in these 4*x* × 4*x* crosses exhibited higher frequencies of seed production and better growth than the 6*x* progeny. These families exhibited lower vigor than 100% 4*x V. corymbosum* material but were dramatically superior to the previously generated 6x hybrids.

In support of the first set of results, we found the crosses of 4*x* US 1930 × 6*x V. virgatum* produced septaploid hybrids, the expected result of a fusion of a 2*n* = 4*x* = 48 egg from the hybrid with a 1*n* = 3*x* = 36 pollen nucleus from *V. virgatum*. These hybrids had the expected intermediate morphology, as well as reasonable fertility and improved fruit size over US 1930. Our limited reciprocal crosses of 6*x* ‘Nocturne’ × 4*x* US 1930 produced a small number of verified pentaploids, parallel to the expected results seen from the functioning of normally reduced gametes seen in 6*x* × 4*x* crosses. These 5*x* hybrids, although vigorous, exhibited morphology that was less clearly hybrid than the 7*x* hybrids seen in the reciprocal cross.

Although our initial goal was to bridge this germplasm to 4*x V. corymbosum*, the 4*x* F_1_ × 6*x* jump to 7*x* ploidy level is a viable alternative for introgression of *V. meridionale-V. vitis-idaea* germplasm into rabbiteye material, especially since the 7*x* hybrid materials possess a modicum of fertility due to polyploid buffering. Additionally, these 7*x* hybrids also have a better level of vigor than the 6*x* derived from 4*x* F_1_ × 4*x* highbush crosses due to the invigorating nature of rabbiteye (*V. virgatum*) germplasm.

The crosses of US 1930 in reciprocal combinations with 6*x V. virgatum* allow the potential bridging of *V. meridionale-V. vitis-idaea* to cultivated *V. virgatum* in multiple ways: via the 5*x* second generation hybrids (through 6*x V. virgatum* × 5*x* crosses) and via the 7*x* second generation hybrids (via 7*x* × 6*x V. virgatum* crosses). Crosses of 6*x V. virgatum* × 7*x* hybrids also seem feasible.

In considering a reasonable explanation for the outcomes of our primary crosses, we examined concepts of genome strengths and endosperm development. Johnston and Hanneman [15] postulated an integral system denoting genome strengths, termed endosperm balance number (EBN), wherein species with matching EBN numbers have the potential to produce interspecific hybrids regardless of parental ploidy and regardless of the possibility of odd-ploid offspring. Ehlenfeldt and Hanneman [16], in investigating genetic control of the endosperm balance number system in *Solanum*, documented asymmetric results in reciprocal backcrosses of experimentally produced, interspecific, intermediate-EBN hybrids, with both their higher and lower EBN species parents. The difference in female and male strengths in the developing endosperm resulted in different levels of viable seed set and different distributions of seed sizes. The directionality of these crosses in *Solanum* reflected earlier studies of 2*x*–4*x* crosses and triploid block with regard to relative dosages of female and male genes in the developing endosperm [17,18]. Crosses in female-excess directions produced higher levels of viable seed with many seeds being reduced in size. Crosses in male-excess directions produced fewer viable seeds with a high frequency of oversize aborted types. Those results were used to construct a quantitative three gene model; however, in those crosses, all of the parents, F_1_ hybrids, and backcross progeny existed at a single diploid ploidy level. Other *Solanum* studies demonstrated the success of 2*n* gametes in bridging interspecific barriers by 2*n* + 1*n* gametic combinations [19]. Our results with respect to asymmetry may be reflective of similar dosage effects; however, we have not been able, thus far, to fit our results to any encompassing quantitative model.

At some level, our crosses suggest that the genome strength expression of *V. meridionale* (or as a somewhat more remote possibility, *V. vitis-idaea*) is different when used as female versus male. It is an interesting concept, in which one must consider how it would affect self-pollination. Endosperm developmental control/genomic strength is thought to be the result of imprinting, but it is unclear if the possibility exists for asymmetric imprinting of female and male genome strengths. We believe based upon our results that there would be no bar to differential imprinting of male and female genome strengths relative to other species. If the species had evolved in such a manner, such a difference would only be visible and manifest in crosses with other species and ploidies, as in our results. However, to fully comprehend, recognize, and prove this possibility, one would need to monitor cross-directionality, ploidies, fertility, stylar interactions, degree of seed development, and general success/failure outcomes. For any such species, a system of asymmetric crossing success might be an evolutionary system that would critically regulate interspecific gene flow between it and congeneric species.

We know from other *V. meridionale* research that 4*x V. corymbosum* × 4*x V. meridionale* crosses succeed, suggesting that *V. meridionale* (as male) and *V. corymbosum* (as female) have relatively comparable genome strengths. However, limited unemasculated crosses of 4*x V. meridionale* × 4*x V. corymbosum* have set seed but failed to produce any unambiguous hybrids, and all offspring have appeared to be *V. meridionale* selfs. Insufficient studies have been done to fully understand why. The failure of these 4*x V. meridionale* × 4*x V. corymbosum* crosses is suggestive, but not at this point conclusive, that 4*x V. corymbosum* (as a male) may have a genomic strength greater than *V. meridionale*, resulting in male-excess type cross failure.

*V. meridionale* has shown itself to be a highly unusual species that can produce 4*x* hybrids with the commercial *Vaccinium* species, *V. vitis-idaea*, *V. corymbosum*, and *V. macrocarpon* [4,5,6]. It has also hybridized with various other 2*x* and 4*x* materials [3]. We are only beginning to understand the possibilities of using *V. meridionale* both in terms of its crossing behavior and its value. Within this material, much more needs to be determined about the fertility of primary and backcross hybrids before we can judge the true nature of its crossing system, but we believe the results from the crosses reported here will help map our path forward in the use of these materials.

## 4. Materials and Methods

### 4.1. Plant Materials

The genotypes used and their compositions and origins are listed in Table 4. Species compositions were derived using published pedigree analyses [20,21,22].

### 4.2. Pollinations

For all materials, pollen was extracted from open flowers by manual manipulation, and collected on glassine weighing paper. Pollen was stored for up to a month under refrigerated and desiccated conditions until used for pollination.

Because of the small and somewhat delicate nature of the flowers on many of the parents, no emasculation was used. All pollinations were performed in an insect-free greenhouse, and it was presumed that hybrids would be morphologically recognizable. Pollinations were made on mature, open flowers using a graphite pencil tip dipped into the collected pollen, then applied to the stigmas of unemasculated flowers. Number of pollinations varied depending upon flower availability.

### 4.3. Seed Extraction and Germination

Pollinations and fruit set were recorded. Fruit was collected when ripe and measured for fruit size (mm) at the time of seed extraction. Extraction was performed manually under a dissecting microscope, and the seeds were evaluated for number and quality. For our purposes, seed were classified as: good, good–fair (g-f), fair, fair–poor (f-p), poor, or aborted. ‘Good’ and ‘fair’ described seed that subjectively ranged from those considered fully normal to those somewhat reduced in size and/or development but nonetheless were judged likely to be capable of germination. ‘Poor’ described seed which displayed reduced size and/or development, often flattened or brown, and judged less likely to be capable of germination. Intermediate ratings were used as needed. ‘Aborted seed’ were those that were flat and brown and generally translucent. Subjective observations were made of the size and quality of aborted seed. In subsequent summaries, the categories good, good–fair, and fair were considered as viable seeds.

Seeds were germinated on a greenhouse mist bench in a soil mix composed of 50:50, peat:sand mixture. At approximately a 3 true-leaf stage, seedlings were transplanted to 36-cell flats. Subsequently plants were transferred to larger pots as appropriate for their growth stage. When actively growing, plants were maintained in an insect-free greenhouse maintained at 18 °C or above. For over-wintering, plants were grown in a greenhouse maintained between 0–4.5 °C to avoid freezing and to optimize accumulation of floral-bud chilling units.

### 4.4. Ploidy Determinations

As plants achieved a size at which it was felt foliage could be sacrificed, tissue was collected for flow cytometry. For cytometry measurements, sampled leaf material (1 cm^2^/20 to 50 mg) together with leaf material of an internal standard with known DNA content (*Zea mays* L.) was chopped with a sharp razor blade in 0.5 mL of extraction buffer (CyStain PI absolute P buffer, catalog number 05-5502; Partec, Münster, Germany) containing RNAse, 0.1% dithiothreitol (DTT), and 1% polyvinylpyrrolidone (ice cold) in a plastic petri dish. After 30 to 60 s of incubation, 2.0 mL staining buffer (CyStain PI absolute P buffer) containing propidium iodide (PI) as fluorescent dye, RNAse, 0.1% DTT, and 1% polyvinylpyrrolidone was added. The sample, containing cell constituents and large tissue remnants of the chopped leaves, was then filtered through a 50 mm mesh nylon filter. After an incubation of at least 30 min at room temperature, the filtered solution with stained nuclei was measured with the flow cytometer [CyFlow ML (Partec) with a green diode laser 50 mW 532 nm (for use with PI). Software was Flomax Version 2.4 d (Partec)]. The DNA amount of the unknown samples was calculated by multiplying the DNA amount of the internal standard with the DNA ratio of the relative DNA amount of the unknown sample and the internal standard. DNA amounts were measured and were compared to a set of standards covering a diploid to hexaploid range (2*x V. darrowii* ‘Fla 4B’, 4*x V. corymbosum* ‘Duke’, and 6*x V. virgatum* ‘Powderblue’) to determine basic ploidy levels.

## Figures and Tables

**Table 1:** Crosses of 4*x V. meridionale*—*V. vitis-idaea* hybrids (US 1930, US 1933, US 1993) as females with 4*x* males, and an advanced generation 6*x* hybrid (US 2386) as female with 4*x* and 6*x* males.

					Seed Quality					Viable			No. of		
			Poll.	Fruit	Good	g-f	Fair	f-p	Poor	Seed ^z^	s/poll ^y^	s/f ^x^	Plants	Ploidy	Notes
**4*x* F_1_ Hybrids *×* 4*x V. corymbosum* and Other 4*x* Selections**															
F_1_ US 1930	×	4*x V. corymbosum* ARS 99-72	43	8	10	9	1	4	1	20	0.5	2.5	-		
“ ”	×	‘Cara’s Choice’	47	28	6	3	9	5	1	18	0.4	0.6	2	6*x*	low vigor
“ ”	×	‘Dixieblue’	45	31	12	12	9	8	190	33	0.7	1.1	3	5*x*, 5.75*x*, 5*x*	“
“ ”	×	‘Magnolia’	17	15	8	14	9	4	-	31	1.8	2.1	-		
“ ”	×	4*x* F_1_ US 1896	32	27	14	24	9	16	207	47	1.5	1.7	3	6*x*	low vigor
“ ”	×	4*x V. corymbosum* US 2117	15	*	*	*	*	*	*	-	-	-	1	6*x*	“
“ ”	×	4*x V. padifolium* US 908	40	16	23	8	19	28	27	50	1.3	3.1	1	6*x*	US 2532, low vigor
“ ”	×	‘Sweetheart’	*	*	*	*	*	*	*	-	-	-	1	6*x*	low vigor
F_1_ US 1933	×	‘Camellia’	16	8	10	1	-	-	-	11	0.7	1.4	4	6*x*	“
“ ”	×	‘Sweetheart’	30	*	*	*	*	*	*	-	-	-	1	6*x*	“
F_1_ US 1993	×	‘Sweetheart’	9	*	*	*	*	*	*	-	-	-	1	6*x*	US 2386, low vigor
		Total	**294**	**133**						**210**	**avg.**	**avg.**	**17**		
											**0.71**	**1.6**			
**2nd generation 6*x* F_1_ *×* 4*x V. corymbosum***															
US 2386	×	‘Camellia’	19	18	249	3	10	-	-	262	13.8	14.6	<170	20 sampled @ 5*x*	medium vigor
“	×	‘Chandler’	19	16	119	-	1	-	2	120	6.3	7.5	not planted	-	
		Total	**38**	**34**						**382**	**avg.**	**avg.**			
											**10.05**	**11.2**			
**2nd generation 6*x* F_1_ *×* 6*x V. virgatum***															
US 2386	×	ARS 07-97	**4**	**1**	28	-	-	1	-	**28**	**7.0**	**28.0**	16	not determined	good vigor

* Data lost. ^z^ Seeds considered viable were those in the categories good, good–fair, and fair. ^y^ Seed/pollination. ^x^ Seed/fruit.

**Table 2:** Crosses of 4*x V. meridionale*—*V. vitis-idaea* hybrids (US 1930, US 1993) as females with 6*x* males, and an advanced generation 7*x* hybrid (US 2521-A) as female with 6*x* males.

					Seed Quality					Viable			No. of		
			Poll.	Fruit	Good	g-f	Fair	f-p	Poor	Seed ^z^	s/poll ^y^	s/f ^x^	Plants	Ploidy	Notes
**4*x* F_1_ Hybrid *×* 6*x V. virgatum* and Other 6*x* Selections**															
F_1_ US 1930	×	ARS 07-97	36	36	11	-	1	-	-	12	0.3	0.3	-		
“ ”	×	ARS 16-57	23	1	2	-	-	-	-	2	0.1	2.0	-		
“ ”	×	‘Baldwin’	27	21	14	1	-	-	8	15	0.6	0.7	2	7*x*	US 2522-#
“ ”	×	‘Delite’	14	12	4	1	-	-	-	5	0.4	0.4	-		
“ ”	×	‘Florida Rose’	29	26	8	1	2	1	2	11	0.4	0.4	-		
“ ”	×	‘Pink Lemonade’	34	24	16	2	4	1	-	22	0.6	0.9	-		
“ ”	×	‘Powderblue’	26	8	3	-	6	-	-	9	0.3	1.1	-		
“ ”	×	US 2320	58	30	14	-	1	-	-	15	0.3	0.5	6	7*x*	US 2521-#
“ ”	×	US 2321	50	22	7	1	2	-	-	10	0.2	0.5	-		
F_1_ US 1993	×	‘Powderblue’	36	1	1	-	-	-	-	1	0.03	1.0	1	7*x*	US 2523
		Total	**333**	**181**						**102**	**avg.**	**avg.**	**9**		
											**0.31**	**0.6**			
**2nd generation 7*x* (=4*x* F_1_ hybrid *×* 6*x V. virgatum*) *×* 6*x V. virgatum***															
7*x* US 2521-A	×	‘Florida Rose’	8	3	3	3	0	0	0	6	0.8	2.0			
“ ”	×	US 2320	8	6	47	0	2	2	0	49	6.1	8.2			
“ ”	×	US 2328	9	9	27	0	1	2	0	28	3.1	3.1			
		Total	**25**	**18**						**83**	**avg.**	**avg.**			
											**3.3**	**4.6**			

^z^ Seeds considered viable were those in the categories good, good–fair, and fair. ^y^ Seed/pollination. ^x^ Seed/fruit.

**Table 3:** Crosses of 4*x V. meridionale*—*V. vitis-idaea* hybrids (US 1930, US 1933) as males with 4*x* and 6*x* female genotypes and crosses of an advanced generation 4*x* hybrid (US 2537-A) with 4*x V. corymbosum* selections.

					Seed Quality					Viable			No. of		
			Poll.	Fruit	Good	g-f	Fair	f-p	Poor	Seed ^z^	s/poll ^y^	s/f ^x^	Plants	Ploidy	Notes
**4*x V. corymbosum* Materials *×* 4*x* F_1_ Hybrids**															
‘Camellia’	×	F_1_ US 1930	24	22	2	3	-	-	-	5	0.2	0.2	0	-	
‘Cara’s Choice’	×	“ ”	11	11	2	2	2	3	15	6	0.5	0.5	21	4*x*	medium vigor
‘Sharpblue’	×	“ ”	12	6	2	33	5	1	14	40	3.3	6.7	24	4*x*	US 2537-A, medium vigor
US 2117	×	“ ”	7	6	11	10	2	-	-	23	3.3	3.8	12	not determined	
“	×	F_1_ US 1933	25	22	2	10	23	5	-	35	1.4	1.6	12	not determined	
		Total	**54**	**45**						**109**	**avg.**	**avg.**	69		
											**2.02**	**2.4**			
**6*x V. virgatum* materials *×* 4*x* F_1_ hybrids**															
‘Nocturne’	×	F_1_ US 1930	72	30	40	-	-	1	1	40	0.6	1.3	0		
“	×	F_1_ US 1933	7	3	9	-	-	-	1	9	1.3	3.0	2	5*x*	vigorous
US 2320	×	F_1_ US 1930	17	11	3	-	-	-	-	3	0.2	0.3	0		
		Total	**89**	**41**						**52**	**avg.**	**avg.**	2		
											**0.58**	**1.3**			
**2nd generation (4*x V. corymbosum ×* F_1_ hybrid) × 4*x V. corymbosum***															
US 2537-A	×	‘Duke’	8	8	81	6	7	7	8	94	11.8	11.8			germinating
“	×	‘Magnolia’	9	7	133	18	13	4	21	164	18.2	23.4			germinating
“	×	‘Talisman’	20	11	34	2	4	2	21	40	2.3	3.9			germinating
		Total	**37**	**26**						**298**	**avg.**	**avg.**			
											**8.05**	**11.5**			

^z^ Seeds considered viable were those in the categories good, good–fair, and fair. ^y^ Seed/pollination. ^x^ Seed/fruit.

**Table 4:** Plant genotypes utilized in hybridization experiments.

Species and Genotype		Species Composition (%) ^z,y^ and Reference
4*x V. meridionale-V. vitis-idaea* S_1_ hybrids		
	US 1930, US 1933, US 1993	50 *merid*, 50 *v-i* [4]
4*x V. corymbosum* cultivars and other 4*x* germplasm		
	ARS 99-72 (=‘Elliott’ × ‘Bluegold’)	90 *cor*, 7 *ang*, 3 unk, USDA sel’n.
	‘Camellia’	72 *cor*, 20 *dar*, 4 *vir*, 2 *ang*, 3 unk [23]
	‘Cara’s Choice’	48 *cor*, 20 *dar*, 15 *vir*, 15 *con*, 2 *ang* [24]
	‘Dixieblue’	71 *cor*, 25 *dar*, 4 *ang* [25]
	‘Duke’	96 *cor*, 4 *ang* [26]
	‘Magnolia’	77 *cor*, 10 *dar*, 8 *vir*, 6 *ang*, 1 *ten* [27]
	‘Sharpblue’	42 *cor*, 29 *dar*, 15 *vir*, 2 *ang*, 13 unk [28]
	‘Sweetheart’	67 *cor*, 16 *dar*, 12 *ang*, 5 unk, <1 *ten* [29]
	‘Talisman’	88 *cor*, 5 *dar*, 3 *ang*, 4 *vir* [30]
	US 908 (4*x V. padifolium*)	100 *pad* [31]
	US 1896 (=US 908 × US 1825)	50 *pad*, 30 *cor*, 12 *dar*, 3 *vir*, 2 *ang*, 3 unk [31]
	US 2117 (=‘Cara’s Choice’ × US 1116)	67 *cor*, 11 *dar*, 8 *vir*, 8 *con*, 3 *ang*, 4 unk, <1 *ten*, USDA sel’n.
	US 2537-A (=‘Sharpblue’ × US 1930)	*see parents*, USDA breeding selection
6*x V. virgatum* cultivars and other 6*x* germplasm		
	ARS 07-97 (=T 451 × ‘Nocturne’)	75 *vir*, 13 *con*, 13 *cor*, <1 *dar/ten/ang*/unk, USDA sel’n.
	ARS 16-57 (=T 451 × ARS 08-176)	75 *vir*, 25 *con*, USDA sel’n.
	‘Baldwin’	100 *vir* [32]
	‘Delite’	100 *vir* [33]
	‘Florida Rose’	100 *vir* [34]
	× ‘Nocturne’	50 *vir*, 25 *con*, 25 *cor*, <1 *dar/ten/ang*/unk [35]
	× ‘Pink Lemonade’	50 *vir*, 50 *cor*, <1 *dar/ten/ang*/unk [36]
	‘Powderblue’	100 *vir* [37]
	US 2320 (=‘Nocturne’ × ‘Florida Rose’)	*see parents*, USDA breeding selection
	US 2321 (=‘Nocturne’ × ‘Florida Rose’)	*see parents*, USDA breeding selection
	US 2386 (=US 1993 × ‘Sweetheart’)	*see parents*, USDA breeding selection

^z^ Compositional values were rounded to the nearest whole percent. Totals may be slightly lower or higher than 100%. ^y^ *ang* = *V. angustifolium*, *con* = *V. constablaei*, *cor* = *V. corymbosum*, *dar* = *V. darrowii*, *mer* = *V. meridionale*, *ten* = *V. tennellum*, *v-i* = *V. vitis-idaea*, *vir* = *V. virgatum*, unk = unknown.

## Data Availability

All critical data regarding this study are presented within this article.
