# Correction: Ehlenfeldt et al. Asymmetric Reciprocal Crossing Behavior of an Andean Blueberry (*V. meridionale*) × Lingonberry (*V. vitis-idaea*) Hybrid. *Plants* 2022, *11*, 3152

**DOI:** 10.3390/plants12152864

**Published:** 2023-08-04

**Authors:** Mark K. Ehlenfeldt, Elizabeth Ogden, Lisa J. Rowland

**Affiliations:** 1 Philip E. Marucci Center for Blueberry and Cranberry Research and Extension, USDA-ARS, Chatsworth, NJ 08019, USA; 2 Genetic Improvement of Fruits & Vegetables Laboratory, USDA-ARS, Beltsville, MD 20705, USA; elizabeth.ogden@usda.gov (E.O.); jeannine.rowland@usda.gov (L.J.R.)

In the original publication [1], there was a mistake in format of the Tables 1–3. The original publication has been updated. The authors state that the scientific conclusions are unaffected.

## References

[1] Ehlenfeldt M.K., Ogden E., Rowland L.J. (2022). Asymmetric Reciprocal Crossing Behavior of an Andean Blueberry (*V. meridionale*) × Lingonberry (*V. vitis-idaea*) Hybrid. Plants.

